# Experimental Analysis of the Correlation Between Cutting Parameters and Recurrence Measures During End Mill Flute Grinding

**DOI:** 10.3390/ma18235284

**Published:** 2025-11-23

**Authors:** Krzysztof Kęcik, Marcin Sałata

**Affiliations:** 1 Department of Applied Mechanics, Faculty of Mechanical Engineering, Lublin University of Technology, 20-618 Lublin, Poland; 2 Department of Manufacturing Techniques and Automation, Faculty of Mechanical Engineering and Aeronautics, Rzeszow University of Technology, 35-959 Rzeszów, Poland; msalata@prz.edu.pl

**Keywords:** grinding, flute, recurrence, stability, mathematical model, dominant index

## Abstract

This paper presents a comprehensive analysis of the flute grinding process in end cutters, focusing on the influence of machining parameters on recurrence indicators. Recurrence quantification analysis (RQA) was employed to assess the dynamic behavior, regularity, and predictability of the process. Based on experimental data, a grinding force model was developed, along with mathematical formulations of the recurrence indicators. Leveraging these models, a novel parameter, the dominant index was proposed to identify which cutting parameters have the greatest impact on the RQA measures. The results reveal that cutting speed exerts a dominant influence on both the grinding force and recurrence metrics. However, for specific indicators, the feed rate emerges as the prevailing factor. The study also demonstrates a strong correlation between cutting parameters and the harmonic content of the grinding force signal. Furthermore, increasing cutting speed was found to generally stabilize the process, whereas variations in feed rate may either enhance or deteriorate machining stability.

## 1. Introduction

Machine components employed across various industrial sectors typically demand precise shaping [1] and surface preparation [2] to meet functional and quality requirements. Milling is a key process in modern manufacturing, widely used for precise and efficient material removal across various industries. Its capability to produce complex geometries, smooth surfaces, and tight dimensional tolerances makes it indispensable in the production of components for aerospace, automotive, medical, and tooling applications. Among the different milling methods, end milling is particularly valued for its flexibility in machining intricate profiles and its suitability for advanced materials such as polymer matrix composites and high-performance alloys [3,4].

The most commonly used materials for end mills are cemented carbide, high-speed steel, and tool steels with physical vapor deposition (PVD) or chemical vapor deposition (CVD) coatings. A critical feature of an end mill is its flutes, which are precisely formed through grinding operations, typically using specialized grinding wheels to achieve the desired geometry and surface finish. The chip flutes are designed to efficiently evacuate machining chips, reduce cutting forces, and enhance heat dissipation. The geometric configuration of the flutes is essential for ensuring optimal cutter performance and long tool life. The manufacturing of such cutters is a complex and demanding process, typically carried out through precision grinding. Nevertheless, recent research continues to advance the reliability, accuracy, and performance of CNC-based flute grinding technologies.

Li et al. [5] proposed a two-pass grinding strategy for end mill flutes with segmented helix angles, achieving optimized cutting-edge geometry through precise control of grinding wheel positioning and the implementation of advanced posture control algorithms. Microcrack formation during end mill flute grinding was investigated in [6], where the authors employed recurrence analysis to identify and characterize the defects. The problem of grinding milling cutters was investigated by Wang et al. [7]. The authors developed a grinding force model and carried out the design and optimization of the cutter. The mathematical model of grinding forces demonstrated that the selected grinding parameters did not induce edge fracture. The grinding damage mechanism of the cutting edge was predominantly governed by brittle removal, whereas the helix flute experienced a combination of plastic and brittle removal mechanisms. Article [8] presented a method for designing grooves in cutters with variable pitch and helix angle, taking into account the practical feasibility of the manufacturing process. Optimization of the groove structure was proposed to increase cutting efficiency and enhance the surface quality of the machined parts. Moreover, in [9], authors developed a mathematical model of the grinding process for end mills with helical cutting edges, taking into account both the tool geometry and the trajectory of the grinding wheel. The proposed model was implemented in a CAD/CAM simulation environment, enabling precise reconstruction of the blade profile and prediction of machining errors. The results offer valuable guidance for optimizing the grinding process with respect to geometric accuracy and surface quality of cutting tools. Zheng et al. [10] introduced a novel method for grinding the flutes of conical milling cutters. The authors developed an approach for accurately calculating the flute profiles of complex geometries by deriving the conical cutting edge line and determining the grinding wheel’s position throughout the process, based on the design requirements for the rake angle and core radius. Subsequently, a calculation method for modeling complex flute profiles was applied and validated. Ren et al. [11] presented a precise method for grinding grooves in cylindrical cutters using standard 1V1/1A1 grinding wheels. The method utilizes a system of nonlinear equations to accurately determine the grinding wheel’s position throughout the groove grinding process, accounting for all three essential groove parameters. The influence of technological parameters on temperature, grinding forces, and surface roughness during the single-pass grinding of carbide end mill flutes was systematically investigated in [12]. The study showed that cutting parameters significantly affect the thermal, mechanical, and surface properties in grinding.

This paper differs from previous studies in three main aspects. First, while earlier work applied recurrence analyis to cutting processes, it mainly characterized signal complexity rather than modeling a direct relationship between cutting parameters and recurrence measures [13,14,15]. Our approach explicitly captures this dependency, offering deeper insight into process dynamics. Second, the proposed RQA-based model estimates recurrence metrics as functions of cutting parameters and supports predictive assessment of grinding process stability and periodicity. Third, we introduce the Dominant Index (DI) as a novel sensitivity metric that clearly identifies which parameter most strongly influences grinding forces. The integration of DI with recurrence analysis is new, while confirming known trends, such as feed rate effects validates the approach. By combining DI with established sensitivity measures, we provide a robust tool for analyzing and interpreting machining dynamics.

## 2. Methodology and Experiment

### 2.1. Experimental Setup for Grinding of Cutter

**Figure 1:**
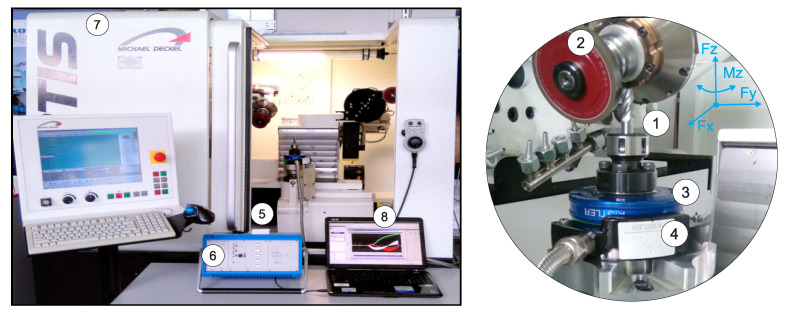
Laboratory setup for the creep-feed flute grinding process: 1. ground cutter, 2. diamond grinding wheel, 3. Kistler rotary dynamometer (type 9123), 4. stationary receiver, 5. A/C converter (type 6009), 6. Kistler amplifier, 7. FORTIS five-axis grinding center, 8. computer.

A dedicated experimental setup for investigating the flute grinding of solid carbide end mills was implemented on the FORTIS five-axis grinding center manufactured by ISOG Technology GmbH, Weilheim, Germany (Figure 1). This advanced CNC grinding machine is designed for both the production and reconditioning of various cutting tools, including drills, milling cutters, taps, and tools with complex or custom profiles. The vertical grinding center features five axes (three translational and two rotational) enabling full-axis interpolation and precise control over tool geometry.

The experimental setup, implemented on a five-axis grinding machine (7), comprises a grinding spindle equipped with a diamond grinding wheel (2), used for machining the cutter (1). A dynamometer (3) is employed to securely hold the workpiece while simultaneously measuring the grinding forces. Additional components (4, 5, 6, and 8) form part of the integrated measurement system. Grinding forces were recorded using a Kistler rotary dynamometer (model 9123, Kistler Group, Winterthur, Switzerland), a multi-channel piezoelectric sensor capable of capturing three orthogonal force components and torque across two selectable measurement ranges. To ensure high measurement accuracy, the fine range was selected, with the following limits: $F_{x}$ and $F_{y}$ from 0 to 450 N, and $F_{z}$ from 0 to 1800 N, according to the axes presented in Figure 1. These values represent the upper boundaries of the fine range as specified by the manufacturer. In this study, all recorded forces remained well below these maximum thresholds. The rotary dynamometer outputs force signals that are digitized via a 12-bit analog-to-digital converter and transmitted wirelessly to a stationary receiver (4). The data is then relayed through a transmission cable to a Kistler 5223B signal amplifier (Kistler Group, Winterthur, Switzerland) (6), where it is converted back into analog form for further processing. The amplified signals are subsequently routed to a National Instruments NI6009 A/C USB converter (5) (Austin, TX, USA), operated via LabView SignalExpress software 2013 V7.0 (Austin, TX, USA), which records the measurement data on a computer (8).

A sampling rate of 5 kHz was applied to ensure sufficient temporal resolution for capturing the dynamic components of the grinding forces. The selected sampling frequency is fully justified from the standpoint of sampling theory, as it substantially exceeds the cutoff frequency of the measurement system (1 kHz). This choice not only satisfies the Nyquist criterion - effectively eliminating the risk of aliasing, but also introduces advantageous oversampling. The increased sampling rate enhances the temporal resolution of the acquired signal and improves the accuracy of both spectral and nonlinear analyses. To preserve the natural characteristics of the signal, no additional digital filtering (such as notch or low-pass filters) was applied. To minimize the influence of coolant spray and environmental disturbances on the force measurements, a custom-designed dynamometer cover was employed. The design and construction of this protective enclosure are described in detail elsewhere [6].

### 2.2. Characteristics of Tool Grinding Process

**Figure 2:**
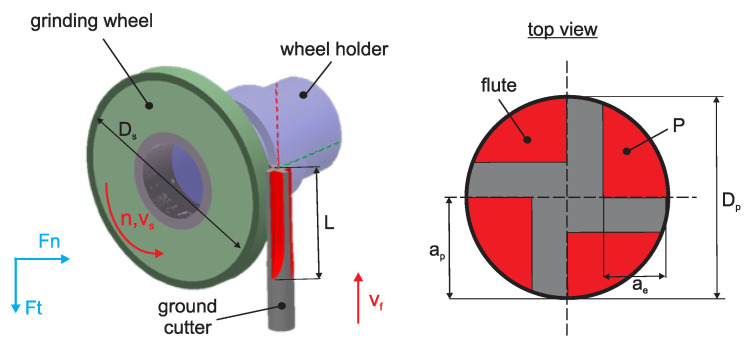
Kinematics of five-axis, single pass flute grinding. $F_{t}$—tangential component of the grinding force, $F_{n}$—normal component of the grinding force, *L*—grinding length, *P*—cut layer cross-sectional area.

The study investigated single-pass grinding of straight flutes in solid carbide end mills made from ultrafine-grained cemented carbide (Figure 2). To minimize the influence of process kinematics, the research specifically focused on straight flute grinding, allowing a direct assessment of the grinding wheel properties. During the single-pass grinding process, the grinding wheel travels at a constant cutting speed $v_{s}$ along a linear path with a feed rate $v_{f}$. The wheel, characterized by a diameter $D_{s}$, engages the workpiece (ground cutter) with a diameter $D_{p}$, generating a grinding width $a_{e}$ and depth $a_{p}$. In this configuration, the entire material allowance represented by the cross-sectional area *P* is removed in a single pass, simultaneously forming a straight flute (shown in red in Figure 2).

The workpiece used in this study was a rod made of TSF22 ultrafine-grained cemented carbide (Ceratizit Group, Mamer, Luxembourg), with a diameter of 12 mm and an h5 tolerance. Within the class of ultrafine-grained carbides, TSF22 offers an excellent balance between hardness and toughness, making it particularly suitable for demanding machining operations. The properties of TSF22 are listed in Table 1. Despite its relatively low cobalt content (8.2%), TSF22 demonstrates outstanding mechanical performance, with a hardness of 1930 HV and a transverse rupture strength of 4400 MPa. For comparison, typical carbide grades exhibit transverse rupture strengths in the range of 3000–4300 MPa.

In the flute grinding tests, type 1A1 diamond grinding wheels (Saint-Gobain Abrasives, Worcester, MA, USA) with a rectangular cross-section were used. The wheel diameter $D_{s}$ was chosen to ensure high grinding speeds while remaining within the spindle’s rotational speed limits. The wheel width *U* was determined by the cross-sectional area of the material to be removed. For the experiments, diamond grinding wheels, with a hybrid bond were selected. This modern bonding system combines synthetic resin with metallic components, with the metallic phase being dominant. According to the manufacturer, variations between different hybrid bonds primarily result from differences in the proportions of resin, metallic fillers, and polymer additives. The diamond grinding wheels used in this study were designated as SP1A1-100-10-10-20 D91SP4400C125. The abrasive grit size, according to FEPA standards (Federation of European Producers of Abrasives, [17]), was 91 μm, and the concentration was 125, corresponding to 5.5 carats per cubic centimeter and representing 31.25% of the abrasive layer volume. Detailed information on the grinding parameters and operating conditions is presented in Table 2.

### 2.3. Research Plan and Parameters

The grinding tests were designed using a structured Design of Experiments (DOE) approach. A face-centered central composite (FCC) design, belonging to the response surface methodology (RSM), was applied to investigate the influence of two independent variables: feed rate ($v_{f}$) and grinding speed ($v_{s}$). The FCC plan employs three levels for each variable (minimum, medium, and maximum), which resulted in nine experimental combinations (Table 3). This design provides the same factor-level structure as a full factorial $3^{2}$ plan while additionally enabling curvature estimation, which is advantageous for subsequent regression modeling and response surface analysis. All remaining process parameters were kept constant to ensure statistical clarity and repeatability of the measured grinding force signals.

A total of nine tests were conducted, including three repetitions for each parameter combination. All repetitions were performed using the same tool to maintain consistent grinding conditions throughout the experimental series. This approach was adopted to minimize variability associated with clamping stiffness, thereby improving the reliability and comparability of the recorded force signals. All other process parameters were kept constant A total of nine experimental tests were carried out, structured to include three repeated trials for each distinct combination of process parameters under investigation. This approach ensured statistical reliability and allowed for the assessment of repeatability in the observed tool wear behavior.

It is worth noting that, beyond cutting forces, parameters such as chip groove roughness and grinding wheel wear are also influenced by the grinding conditions. However, these aspects were not addressed in the present study.

## 3. Method of Analysis

### 3.1. Grinding Force Analysis

Cutting force signals often exhibit stochastic components that cannot be effectively captured using conventional statistical methods. Consequently, the analysis of such nonlinear signals requires the application of more advanced time-series analysis techniques. In this study, both traditional and advanced approaches were employed. For the statistical evaluation of grinding force, the regression model was developed and analyzed. However, traditional statistical approaches often struggle to capture the inherently complex and dynamic nature of process interactions. These methods typically rely on assumptions such as linearity, normality, and stationarity, which may oversimplify the rich and nonlinear characteristics of the systems. For more sophisticated analysis, recurrence plots (RPs) and recurrence quantification analysis (RQA) were applied, which have been shown to be highly effective in characterizing signals generated during cutting processes [14,15,18,19].

In the first stage of the research, the influence of two variable technological parameters, feed rate and grinding speed on the normal component of the grinding force ($F_{n}$) (see Figure 2) was investigated. Subsequently, a regression equation was developed to describe the relationship between the grinding force and the process parameters. This approach enables the estimation of grinding forces under various machining conditions. Additionally, Fast Fourier Transform (FFT) analysis was performed to examine the signal in the frequency domain. This method enables the identification of frequency bands and individual frequency components, along with their respective amplitudes. Such analysis is useful for detecting periodic patterns, resonances, or other anomalies in the signal.

Finally, a comprehensive recurrence analysis was conducted to gain deeper insights into the dynamic behavior of the end mill grinding process. Recurrence diagrams were generated, and the corresponding quantifications were calculated and plotted according to the cutting parameters. Subsequently, mathematical models of these indicators were developed. Based on these models, a new parameter, called the dominant index, was introduced to identify the cutting parameters that most strongly influence the RQA measures.

### 3.2. Recurrence Plot

A recurrence plot (RP) is a graphical tool designed for the analysis of nonlinear signals [20,21,22,23,24]. This method allows the identification of similar states in phase space and is typically based on the reconstruction of the signal using the time-delay embedding method. Based on a single measured signal from the experiment, $x\left(t\right)=\left(x_{1},x_{2},\ldots ,x_{n}\right)$ it is possible to reveal hidden relationships between data points by reconstructing the phase space. This embedding method creates a new delayed vector, $X_{i}$ based on Takens’ theory.

(1) $$X_{i}=\left(x_{i},x_{i+\tau },x_{i+2\tau },\ldots ,x_{i+(m-1)\tau }\right),$$

where τ is the lag, and *m* is the embedding dimension.

Once the time-delay embedding has been applied to the original time series, the recurrence matrix can be derived using Equation (2a) or Equation (2b). This matrix encodes the pairwise distances between state vectors and serves as the basis for constructing the recurrence plot.

(2a) $$R_{i,j}^{\epsilon }=\left\{\begin{array}{cc} 1 & \mathrm{if}\;∥X_{i}-X_{j}∥\leq \epsilon ,\;i,j=1,\ldots ,N.\\ 0 & \mathrm{otherwise} \end{array}\right.$$

(2b) $$R_{i,j}=∥X_{i}-X_{j}∥,\;i,j=1,\ldots ,N.$$

where ||·|| denotes the Euclidean norm used to quantify the distance between recurrent points, *N* is the number of considered points, and ϵ represents the threshold that defines the maximum allowable distance for points to be considered close. In essence, it establishes the proximity criterion within the phase space that determines whether two states are classified as recurrent.

Recurrence plots can be generated using two principal approaches, corresponding to Equations (2a) and (2b). In the first method, a fixed threshold parameter ϵ is introduced to determine whether recurrence occurs: points are considered recurrent if the distance between them does not exceed ϵ. The resulting plot is binary, and recurrent points are marked as black dots, while nonrecurrent points remain unmarked (usually appearing as white space). However, since the choice of ϵ significantly influences the resulting RP structure, an alternative approach avoids thresholding altogether. Instead of a binary representation, a third parameter, most commonly color, is used to encode the actual distance between points. This continuous representation offers a more detailed visualization of recurrence intensity, preserving richer information about the system’s dynamics and revealing subtle patterns that may be obscured in thresholded recurrence plots.

### 3.3. Recurrence Measures

Recurrence plots are useful tools for assessing periodicity and stochasticity in dynamical systems. However, due to their inherently qualitative nature, a precise and objective evaluation can be challenging [25,26]. To address this limitation, recurrence plots are often complemented by advanced numerical and statistical measures Table 4, known as recurrence quantification analysis, which enable a more rigorous and quantitative characterization of the system’s dynamics [27,28,29].

By analyzing the recurrence quantification measures, it is possible to assess whether the examined signal (or process) exhibits stationary, stochastic, or transitional dynamics. A periodic process shows long diagonal lines in RP structure [35]. A recurrence rate (RR) corresponds to the definition of the correlation sum [21]. Determinism (DET) called also predictability, is reflected by the presence of long diagonal lines, indicating that the system is more recurrent and potentially periodic. Similarly, a very high value of the longest diagonal line length ($L_{\max}$) suggests a high degree of predictability in the system’s evolution. Elevated recurrence entropy implies that the underlying dynamics are complex and exhibit a broad distribution of recurrent patterns. On the other hand, low values of laminarity (LAM) and trapping time (TT) are indicative of rapidly changing, non-laminar processes. Furthermore, low values of recurrence time-based measures ($T_{1}$ and $T_{2}$) suggest that the system does not remain in a particular state for long and exhibits a limited number of distinct laminar (stagnant) phases. Shannon entropy ENT measures the level of complexity [21].

## 4. Results

### 4.1. Measured Grinding Force

**Figure 3:**
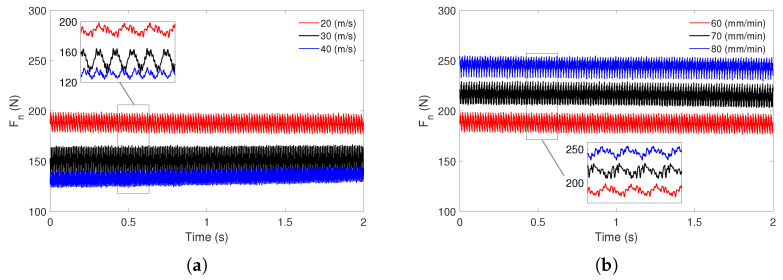
The grinding normal component forces $F_{n}$ for (**a**) $v_{f}$ = 60 mm/min and various $v_{s}$, and (**b**) $v_{s}$ = 20 m/s and various $v_{f}$.

During the experiment, the normal component of the grinding force ($F_{n}$) was continuously measured (see Figure 2). Figure 3a shows the recorded force signals for a constant feed rate and varying grinding speeds. The red, black, and blue curves correspond to grinding speeds of $v_{s}=20$ m/s, $v_{s}=30$ m/s, and $v_{s}=40$ m/s, respectively. An increase in grinding speed leads to a noticeable decrease in the average normal force, accompanied by changes in the oscillatory characteristics of the signal. At the lowest speed of 20 m/s, the force signal exhibits a higher mean value and more pronounced fluctuations. In contrast, at the highest speed of 40 m/s, both the mean force and the amplitude of oscillations are significantly reduced.

Figure 3b presents the evolution of the $F_{n}$ as a function of time for three different feed rates: 60 mm/min (red line), 70 mm/min (black line), and 80 mm/min (blue line). For all tested feed rates, the force signal exhibits oscillatory behavior, reflecting the dynamic nature of the grinding process. As the feed rate increases from 60 mm/min to 80 mm/min, a general trend of increasing average normal force is observed. The red curve (60 mm/min) shows the lowest mean force and relatively moderate fluctuation amplitude. The black curve (70 mm/min) displays a higher mean force and slightly increased oscillation amplitude. The blue curve (80 mm/min) exhibits the highest mean force and the most pronounced force fluctuations.

### 4.2. Statistical Analysis

The regression equation for the normal component $F_{n}$ of the grinding force is presented in Equation (3). In order to analyze the quality of the model fit to the recorded data, statistical parameters were determined and are shown in Table 5. The same table also reports the *p*-values indicating the statistical significance of the model coefficients.

(3) $$F_{n}\left(v_{s},v_{f}\right)=-17.09+4.50v_{f}+3.03v_{s}-0.094v_{f}v_{s}.$$

Analysis of the statistical parameters of the regression models reveals that the obtained standard deviation (SD) is 6.09 N, with an average response value (*y*) of 194.08 N. This yields a coefficient of variation (CV) of 3.14%, indicating a high degree of repeatability and reliability of the experimental results. The coefficients of determination ($R^{\prime 2}$ = 0.972, $R^{2}$ = 0.962, and $R_{p}^{2}$ = 0.937) confirm an excellent fit of the models to the experimental data, a minimal contribution of random error, and strong predictive capability. The signal-to-noise ratio (SNR) is a key metric for assessing the quality and reliability of experimental data and model adequacy. The value of SNR = 29.98 substantially exceeds the recommended minimum value ($SNR_{rec}$ > 4), further validating the adequacy and robustness of the developed models for describing the investigated process [36].

The analysis of variance (ANOVA) demonstrated that all investigated factors exert a statistically significant influence on the response variable (Table 5, lower section). Extremely low *p* values (*p* < 0.0001 for both $v_{f}$ and $v_{s}$, and *p* = 0.0143 for the interaction term $v_{f}v_{s}$) indicate a high level of statistical significance. The elevated values of the *F*-statistics (*F* = 64.05 for $v_{f}$, *F* = 211.39 for $v_{s}$, and *F* = 9.72 for the $v_{f}v_{s}$ interaction) further confirm that the independent variables account for a substantial proportion of the response variability. Consequently, it can be concluded that the developed models exhibit high predictive accuracy, and the selected technological parameters have a pronounced effect on $F_{n}$, the normal component of the grinding force.

**Figure 4:**
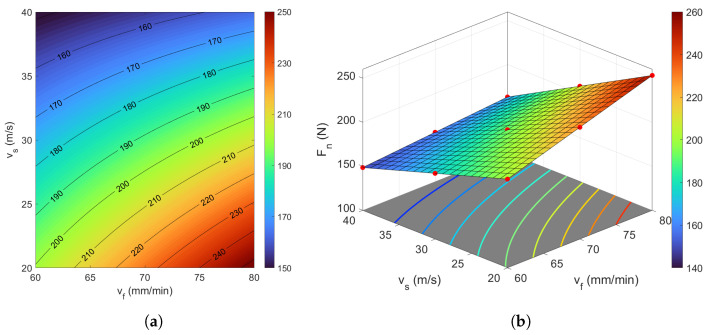
Dependency between the technological parameters $v_{f}$ and $v_{s}$ in relation to the grinding force $F_{n}$: (**a**) contour plot, and (**b**) 3D surface plot. The colorbar represents the magnitude of $F_{n}$.

A graphical interpretation of the developed model is presented in Figure 4a,b. Analysis of the obtained response surfaces indicates that the lowest value of the normal component of the grinding force was 140 N, achieved at the parameters $v_{s}$ = 40 m/s and $v_{f}$ = 60 mm/min. Conversely, the highest value of $F_{n}$ = 255 N was recorded for the parameters $v_{s}$ = 20 m/s and $v_{f}$ = 80 mm/min.

**Figure 5:**
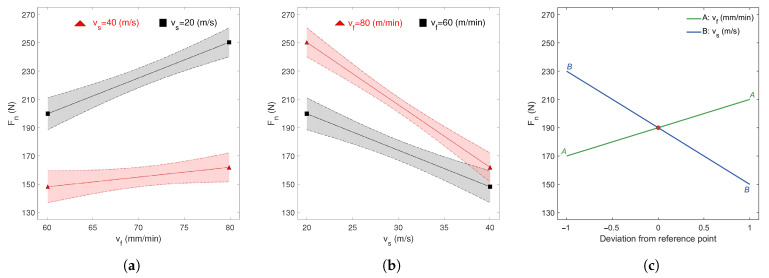
Dependency between the technological parameters $v_{f}$ and $v_{s}$ in relation to the grinding force $F_{n}$: (**a**) feed rate interaction, (**b**) grinding speed interaction, and (**c**) perturbation plot.

To enable a more detailed analysis of the relationship between the technological parameters $v_{s}$ and $v_{f}$ and the normal component of the grinding force, interaction plots were prepared (Figure 5a,b). The interaction analysis of $v_{f}$ as a function of cutting speed $v_{s}$ revealed that an increase in $v_{f}$ leads to an increase in the normal component of the grinding force $F_{n}$, regardless of the value of $v_{s}$ (Figure 5a). This is confirmed by the positive slope of both curves. Additionally, the figure clearly shows a significant two-factor interaction between $v_{f}$ and $v_{s}$. It indicates that at a higher cutting speed ($v_{s}$ = 40 m/s), the influence of feed rate $v_{f}$ on the value of the normal force component $F_{n}$ is less pronounced, as evidenced by the small slope angle of the red curve ($5^{\circ }$). In contrast, at a lower cutting speed ($v_{s}$ = 20 m/s), the effect of feed rate on $F_{n}$ becomes more noticeable, which is confirmed by the larger slope angle of the black curve (about $18^{\circ }$).

Similar relationships were observed in the interaction analysis of cutting speed $v_{s}$ as a function of feed rate $v_{f}$ (Figure 5b). The negative slope of both curves indicates that as the $v_{s}$ increases, the normal component of the grinding force $F_{n}$ decreases. Analysis of the two-factor interaction revealed that at a higher feed rate ($v_{f}$ = 80 mm/min), the influence of $v_{s}$ on $F_{n}$ is more pronounced, as evidenced by a step slope of the red curve ($29^{\circ }$). Conversely, at a lower feed rate ($v_{f}$ = 60 mm/min), the effect of cutting speed on $F_{n}$ remains noticeable, as indicated by the slope of the black curve ($18^{\circ }$), although this influence is less pronounced compared to the effect observed at higher feed rates. A summary of the influence of technological parameters on the grinding force, $F_{n}$, is presented in Figure 5c. The graph illustrates the effects of the feed rate $v_{f}$ and the grinding speed $v_{s}$ on $F_{n}$, as well as their normalized deviations from the central point (0, 0). The reference point corresponds to the central values of the analyzed variables, which are indicated at the top of the graph. Values along the abscissa (deviation from the reference point) are normalized, with −1.0 representing the minimum and 1.0 the maximum values within the experimental range. It can be shown that increasing the grinding speed $v_{s}$ (blue line) leads to a decrease in $F_{n}$, whereas increasing the feed rate $v_{f}$ (green line) causes $F_{n}$ to increase. These observed trends are consistent with previously reported results and analyses. Moreover, examining the slopes of the curves reveals that $v_{s}$ (slope $31^{\circ }$) has a statistically more pronounced effect on the normal component $F_{n}$ than $v_{f}$.

### 4.3. Recurrence Diagrams

Since the analyzed signal is experimental, standardization is usually recommended [37]. In this study, we apply standardization by transforming the data to have zero mean and unit variance (i.e., a mean of zero and a standard deviation of one). This procedure is performed exclusively to remove baseline offsets and reduce the influence of amplitude-related fluctuations, ensuring that the subsequent analysis focuses on the intrinsic temporal structure of the signal rather than its scale or magnitude. An offset in the cutting force signal is observed in our measurements (Figure 3). Additionally, standardization ensures that the cutting force becomes dimensionless. In recurrence analysis, filtering is generally not applied, as the method focuses on uncovering intrinsic patterns and structures within the raw time series data. Preprocessing steps such as filtering may alter the underlying dynamics, potentially affecting the accuracy and interpretability of recurrence-based measures.

**Figure 6:**
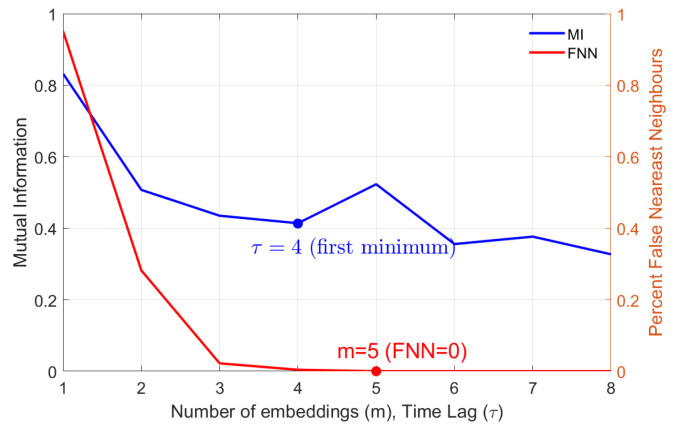
Estimation of embedding dimension *m* (red curve) using the FNN and time lag τ (blue curve) using the MI methods.

To reconstruct the delay vector (Equation (3)) using the embedding method, the first step involves estimating appropriate values for the embedding dimension *m* and time delay τ [31]. The embedding dimension was determined using the False Nearest Neighbors (FNN) method, which evaluates the proportion of falsely identified neighboring points as the dimension *m* increases [38]. The optimal value of mm corresponds to the point at which the number of false neighbors approaches zero. In our case, the optimal embedding dimension was found to be *m* = 5, as indicated by the red marker in Figure 6.

In the subsequent step, the delay parameter τ is determined using the Mutual Information (MI) method [39]. This approach relies on evaluating the amount of mutual information between the original signal and its time-delayed version, thereby identifying the lag that provides the most independent embedding coordinates. In practice, the optimal value of τ is assumed to be the first minimum of the MI criterion [40]. In our analysis, the τ = 4 (blue marker in Figure 6).

**Figure 7:**
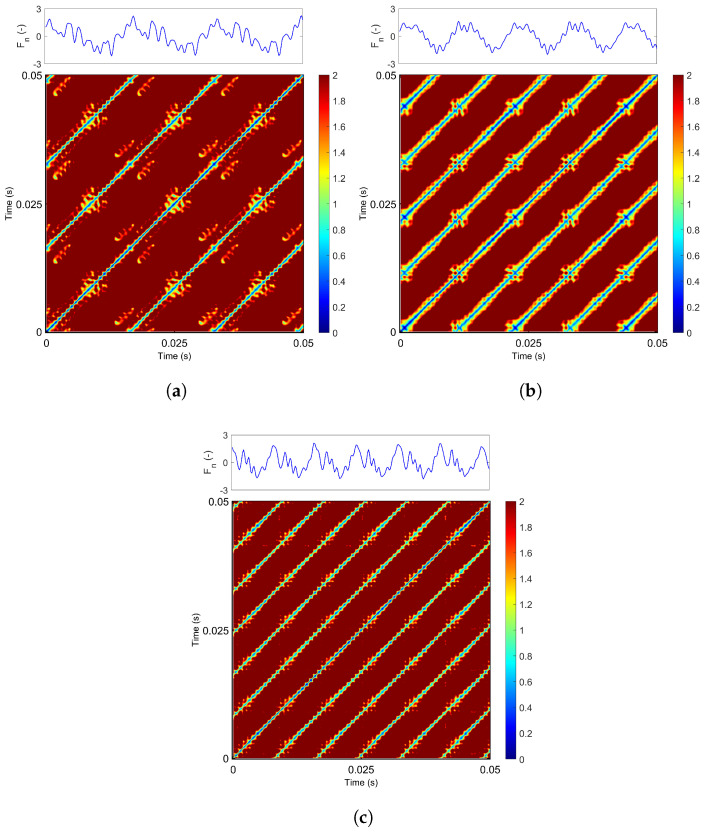
Recurrence diagram of the grinding force component for $v_{f}$ = 60 mm/min and: (**a**) $v_{s}$ = 20 m/s, (**b**) $v_{s}$ = 30 m/s, (**c**) $v_{s}$ = 40 m/s for *m* = 5 and τ = 4. The colorbar indicates ϵ values in the range of 0 to 2.

Representative recurrence plots are presented in Figure 7a–c, obtained for a constant feed rate of 60 mm/min. The upper panels display standardized force signals comprising 500 data points, corresponding to the time window selected for recurrence analysis. The recurrence plots were constructed without imposing a fixed threshold; instead, continuous pairwise distances between time series points were utilized, ranging from 0 (no recurrence) to 2 (high recurrence). It is important to note that the choice of the threshold parameter ϵ is a critical factor in recurrence analysis, as it significantly influences the resulting structures and their interpretation.

At a low grinding speed (20 m/s, Figure 7a), the RP reveals a structure dominated by long blue diagonal lines, indicative of periodic motion. However, interspersed among these lines are irregular clusters of red and yellow points, corresponding to additional harmonic oscillations visible in the force signal. The diagonal lines observed in the recurrence plot represent sequences of recurrent states within the system’s phase space, indicating that the system revisits similar states over time. When the grinding speed is increased to 30 m/s (Figure 7b), the signal exhibits increased regularity and stability. The recurrence plot still shows blue diagonal lines, now accompanied by a subtle yellow and black gradient.

This indicates that the additional oscillations are significantly reduced and more regular, which is also reflected in the smoother grinding force profile shown in the upper panel. A further increase in grinding speed to 40 m/s leads to a noticeable reduction in high-recurrence regions (highlighted in red and yellow, Figure 7c). This indicates that the additional harmonic components of the oscillatory signal are diminishing, resulting in a more regular and partially periodic dynamic behavior of the process.

**Figure 8:**
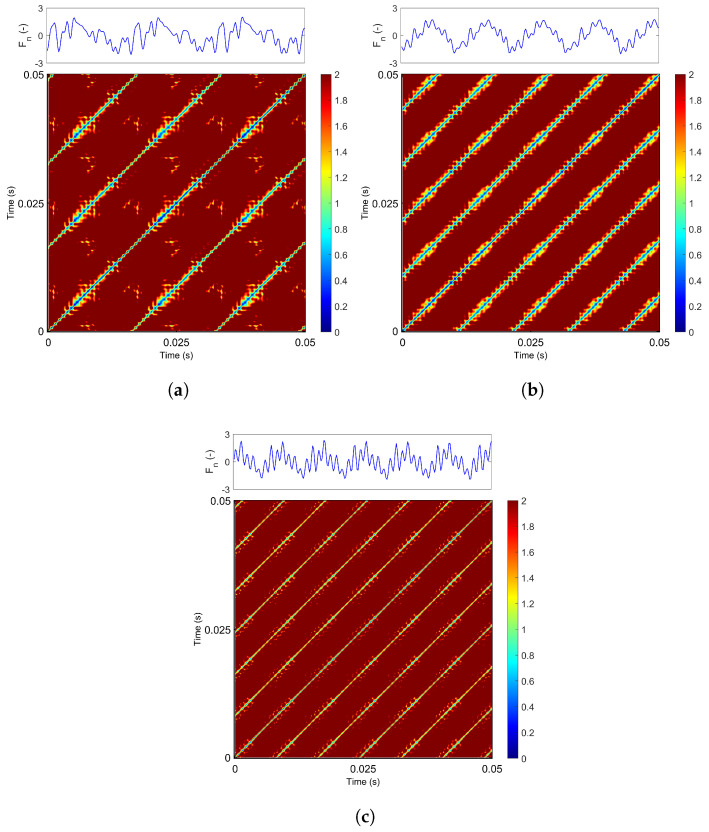
Recurrence diagram of grinding force component for $v_{f}$ = 70 mm/min and: (**a**) $v_{s}$ = 20 m/s, (**b**) $v_{s}$ = 30 m/s, (**c**) $v_{s}$ = 40 m/s for *m* = 5 and τ = 4. The colorbar indicates ϵ values in the range of 0 to 2.

Similar patterns are observed when analyzing RP diagrams at an increased feed rate of 70 mm/min. For a grinding speed of 20 m/s (Figure 8a), the dominant periodic component (represented by the blue diagonal lines) remains largely unchanged. The red and yellow regions appear slightly diminished, while new recurrence points emerge between the diagonal lines, originating from additional harmonics present in the signal. As the grinding speed increases to 30 m/s (Figure 8b), the process again becomes more regular and stable. Long, thicker diagonal lines with yellow and red bands aligned with the periodic pattern are now observed. This effect is also clearly visible in the time-history plot in the top panel.

At a grinding speed of 40 m/s (Figure 8c), the diagonal lines become noticeably thinner, accompanied by additional recurrence structures aligned along the diagonals. The distance between recurrence points decreases (seen in the yellow regions), indicating fewer harmonic components in the system.

**Figure 9:**
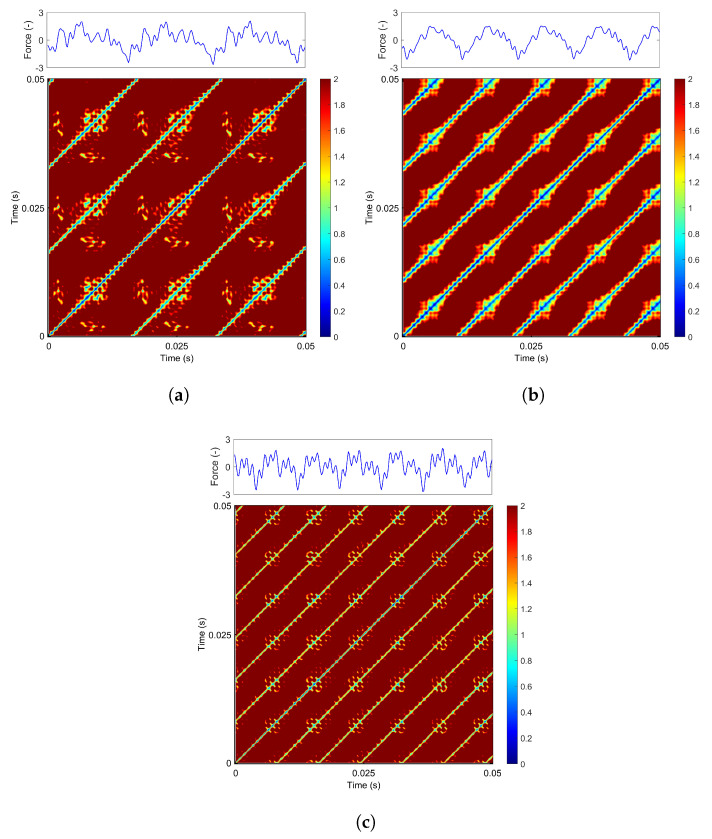
Recurrence diagram of the grinding force component for $v_{f}$ = 80 mm/min and: (**a**) $v_{s}$ = 20 m/s, (**b**) $v_{s}$ = 30 m/s, (**c**) $v_{s}$ = 40 m/s for *m* = 5 and τ = 4. The colorbar indicates ϵ values in the range of 0 to 2.

Finally, for a feed rate of 80 mm/min, the recurrence behavior remains qualitatively consistent with the previously analyzed cases. At a grinding speed of 20 m/s (Figure 9a), the signal exhibits regular periodic motion dominated by a fundamental frequency (visible as blue diagonal lines), accompanied by additional harmonic components. Notably, the red recurrence clusters increase and undergo shape changes. Increasing the grinding speed to 30 m/s (Figure 9b) enhances the periodicity of the signal. The blue lines become more pronounced, while the red and yellow regions consolidate into thicker, well-structured bands. This behavior closely resembles the recurrence patterns observed at the same cutting speed for other feed rates (cf. Figure 7b and Figure 8b), although in this case the addition recurrence regions are larger. However, at a grinding speed of 40 m/s (Figure 9c), the periodicity improves, similar to the previous case. The primary harmonic (blue line) becomes thinner and is interspersed with more yellow regions. It should be noted that this signal exhibits more additional harmonics compared to Figure 7c, as indicated by the increased presence of red and yellow points near the diagonal lines.

In summary, across all analyzed cases, it is observed that the grinding speed has a significant impact on the recurrence plot (RP) structure. The process becomes more periodic, with clearer diagonal lines, and more dominant frequencies appear at a grinding speed of 30 m/s. It should be noted that as the cutting speed increases, the spacing between the blue diagonal lines in the RP decreases, indicating a reduction in the vibration period and a corresponding increase in frequency. The feed rate primarily affects the occurrence of additional recurrence points, while the diagonal lines remain largely unchanged. This indicates that the feed rate mainly influences the presence of additional harmonic components.

**Figure 10:**
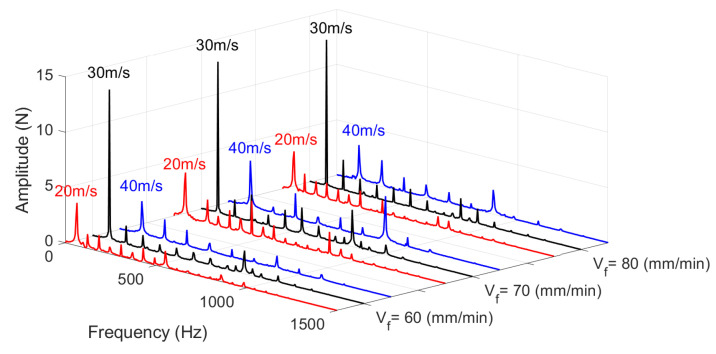
Fast Fourier Transform (FFT) analysis of grinding signals.

For comparison, the Fast Fourier Transform (FFT) of selected grinding forces was performed. This approach allows the analysis to be shifted from the time domain to the frequency domain, providing information about the primary and higher-order frequency components present in the signal (Figure 10). The red line represents the FFT results for a grinding speed of 20 m/s, the black line for 30 m/s, and the blue line for 40 m/s. The results show that at a grinding speed of 30 m/s, the signal exhibits a dominant frequency with an amplitude of approximately 15 N, independent of the grinding feed rate. For speeds of 20 m/s and 40 m/s, the FFT results are similar; however, at the higher speed, the signal contains more harmonic components. Additionally, variations in the feed rate lead to a more even distribution of amplitudes among the harmonics. This effect suggests that changes in feed rate modulate the energy content across a broader range of frequency components, rather than concentrating it around a dominant peak.

As a result, the spectral profile becomes more balanced, with reduced amplitude contrast between the fundamental frequency and its higher-order harmonics. Such redistribution may indicate increased complexity in the force signal and a more heterogeneous dynamic response of the grinding process.

### 4.4. Recurrence Quantification Analysis

Since direct interpretation of recurrence plots is often challenging, numerical methods have been introduced to systematically analyze their structure. These approaches enable quantitative assessment and comparison of individual recurrence measures, commonly referred to as quantifications. The calculations were performed based on the recurrence diagrams shown in Figure 7, Figure 8 and Figure 9, using a threshold value of ϵ=0.4 for all RQA measures. It is worth noting that RQA measures can be influenced by the choice of threshold distance used to construct the recurrence plot. To ensure consistency and comparability across all cases in our analysis, the threshold was kept constant throughout.

**Figure 11:**
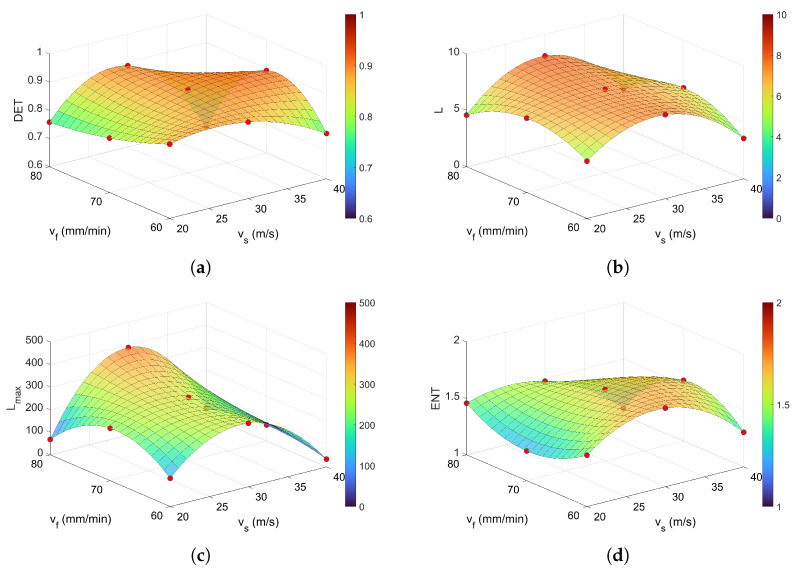
Recurrence quantifications based on diagonal lines: (**a**) DET, (**b**) *L*, (**c**) $L_{max}$ and (**d**) ENT for *m* = 5, τ = 4, and ϵ = 0.4. The colorbar represents the magnitude of the RQA indicator.

Analysis of DET (Figure 11a), derived from diagonal structures in the recurrence plot, reveals values ranging from 0.7 to 0.9. This indicates that the majority of recurrence points contribute to diagonal lines, reflecting a predominantly deterministic dynamic. The average value, $DET_{avg}$ = 0.86, suggests that approximately 86% of the recurrence points form such structures. Further analysis of the average diagonal line length *L* (Figure 11b) shows that lower values, approximately 5, occur at both low and high cutting speeds. In contrast, within the intermediate range of process parameters, *L* increases and stabilizes around 7 (average $L_{avg}$ = 6.76) in the middle of the parameter range. This implies a more deterministic signal behavior, with recurrent patterns persisting over longer time intervals. Higher values of *L* indicate that the system revisits similar states for extended durations. Notably, a similar trend was observed for DET. A similar trend to that observed for *L* is reflected in the behavior of $L_{max}$, although the differences are more pronounced (Figure 11c). For low cutting speeds, as well as at both low and high feed rates, $L_{max}$ reaches a minimum value of approximately 100. In contrast, the longest diagonal line was observed at the highest feed rate $v_{f}$ = 80 mm/min and a moderate cutting speed $v_{s}$ = 20 m/s, where $L_{\max}\approx$ 390. The average value across all conditions was approximately 234. Elevated values of $L_{max}$ indicate extended periods of deterministic behavior, suggesting the presence of stable or cyclic dynamics within the signal. The last diagonal indicator, entropy (ENT), revealed elevated values in the central region of the surface, specifically around $v_{s}=30$ m/s and in the vicinity of $v_{f}$ = 70 mm/min, see Figure 11d. The average value was $ENT_{avg}\approx$ 0.24. A low Shannon entropy indicates that the analyzed signal is more regular and predictable.

**Figure 12:**
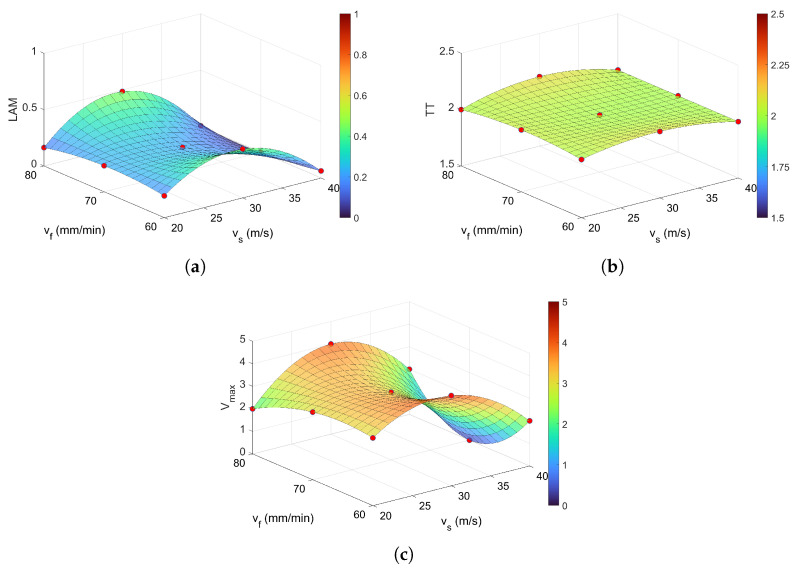
Recurrence quantifications based on vertical lines: (**a**) LAM, (**b**) TT, and (**c**) $V_{max}$ for *m* = 5, τ = 4, and ϵ = 0.4. The colorbar represents the magnitude of the RQA indicator.

The analysis of vertical line structures reveals that the LAM indicator (Figure 12a) follows a trend closely aligned with that of $L_{max}$. The highest LAM values are observed at a cutting speed of $v_{s}$ = 30 m/s and feed rates of $v_{f}$ = 60 mm/min and $v_{f}$ = 80 mm/min, with LAM ≈ 0.4. This indicates that approximately 40% of recurrence points form vertical lines, suggesting a pronounced presence of laminar phases within the system.

For other combinations of cutting parameters, LAM values decrease significantly—typically by a factor of three to four. The average value across all surface measurements is approximately $LAM_{avg}$ ≈ 0.24, indicating that although laminar behavior is present, it is not dominant. The system remains highly dynamic. Low LAM values imply rapid transitions and a lack of prolonged laminar phases, reflecting a system that changes quickly and rarely resides in transitional states. In contrast, higher LAM values point to more stable, laminar behavior. The next recurrence indicator, TT (Figure 12b), exhibits a nearly constant value across the entire range of cutting parameters, with an average of approximately $TT_{avg}\;\approx \;2$. This suggests that the system maintains a consistent level of stability regardless of the specific machining conditions. The longest vertical line, denoted as $V_{max}$, is illustrated in Figure 12c. This measure reflects the maximum duration for which the system remains in the same state. The lowest value of $V_{max}$ is observed at the highest grinding speed ($v_{s}$ = 40 m/s), particularly for a feed rate of $v_{f}$ = 70 mm/min, where it reaches zero (dark blue region in Figure 12c). It is important to note that while TT represents the average length of vertical lines and thus characterizes the general laminar structure of the RP, $V_{max}$ highlights the most extreme cases of laminar behavior, offering a more pronounced indication of prolonged stability within the system.

**Figure 13:**
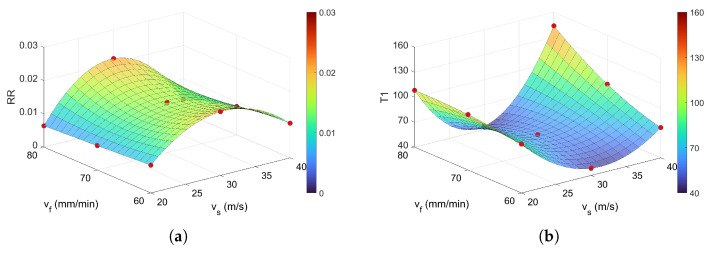

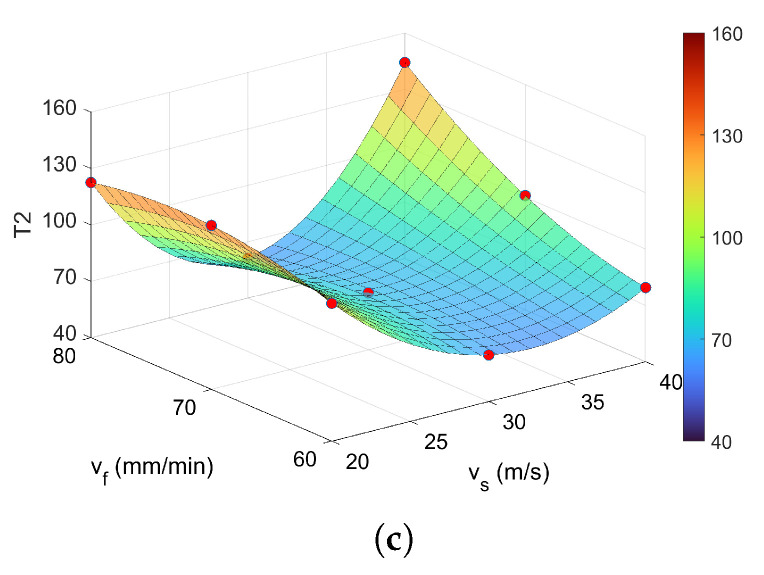
Recurrence quantifications based on recurrence points and distances lines: (**a**) RR, (**b**) T1, and (**c**) T2 for *m* = 5, τ = 4, and ϵ = 0.4. The colorbar represents the magnitude of the RQA indicator.

One of the most important recurrence indicators in the literature is the recurrence rate. It denotes the percentage of recurrent points in the phase space within a certain threshold distance (in our case ϵ = 0.4). Generally, a higher value of RR indicates a high propensity for recurring patterns [21]. A high RR indicates that the system exhibits predictable and structured behavior, with recurring patterns that are sustained over time—indicating a degree of regularity. Analysing the RR surface reveals that lower recurring states (RR below 0.01) occur for low and high grinding speeds ($v_{s}$ = 20 and 40 m/s independent of feed rate (Figure 13a). The average $RR_{avg}$ equals about 0.0135. The shape of the RR surface is similar to DET (Figure 11a), $L_{max}$ (Figure 11c), *L* (Figure 11b) and LAM (Figure 12a).

The final two recurrence measures analyzed are T1 and T2 (Figure 13b,c), which exhibit very similar trends, differing only slightly in their numerical values. The lowest values for both indicators are observed near the grinding speed of $v_{s}$ = 30 m/s, where T1≈60 and T2≈70, respectively. It is important to note that T1 and T2 display inverse behavior compared to other recurrence measures such as DET, *L*, $L_{max}$, and LAM. According to [32], a high value of T1 indicates a more deterministic state, whereas a high value of T2 reflects a more laminar regime. This suggests that, despite differences in formulation, most recurrence indicators lead to consistent conclusions regarding the system’s dynamic behavior.

## 5. Discussion

The recurrence-based findings provide a foundation for developing predictive mathematical models of individual indicators, allowing for precise estimation without the need for extensive experimentation. This is especially important given the high time and resource demands associated with empirical studies in tool production.

To construct a generalized model of recurrence measures, the poly22 fitting option in MATLAB version 24.2.0.2712019 (2024b) is applied. This method corresponds to a second-order polynomial surface approximation involving two independent variables-specifically, the cutting speed and feed rate in the present study. The proposed model enables surface fitting in three-dimensional space, where the response variable is expressed as a function of both inputs through a polynomial equation incorporating linear, interaction, and quadratic terms.

The recurrence indicator RQAm was modeled as a second-order polynomial function of cutting speed $v_{s}$ and feed rate $v_{f}$. This formulation captures both linear and nonlinear effects, as well as their interaction, allowing for a detailed surface analysis of the recurrence behavior across the process parameter space.

(4) $$RQA_{m}\left(v_{s},v_{f}\right)=p_{00}+p_{10}v_{s}+p_{01}v_{f}+p_{20}v_{s}^{2}+p_{11}v_{s}v_{f}+p_{02}v_{f}^{2},$$

where $p_{00}$, $p_{10}$, $p_{01}$, $p_{20}$, $p_{11}$, and $p_{02}$ denote the surface fitting coefficients listed in Table 6. Surface fitting is performed via polynomial regression, which determines the optimal set of coefficients by minimizing the residual sum of squares between the empirical data and the polynomial surface function, with 95% confidence bounds.

Surface fitting is performed using polynomial regression, which determines the optimal set of coefficients by minimizing the residual sum of squares between the empirical data and the polynomial surface function. The resulting coefficients are accompanied by 95% confidence bounds, which indicate the statistical reliability of the fit.

The results obtained from the recurrence model (Equation (4)), developed within the proposed mathematical framework, were superimposed as red markers onto the surfaces presented in Section 4.4. The close agreement between these markers and the numerically computed values of the RQA indicators confirms the model’s predictive accuracy.

To evaluate the quality of the fitted model or recurrence measures, several statistical metrics are calculated, including residuals, root mean square error (RMSE), normalized root mean square error (NRMSE), mean absolute error (MAE), the coefficient of determination (${R\prime }^{2}$), and the adjusted coefficient of determination ($R^{2}$) of the model coefficients Table 7. These measures provide a comprehensive assessment of the model’s accuracy and statistical significance, ensuring the reliability of the derived conclusions.

The regression models applied to the RQA indicators demonstrate varying levels of predictive accuracy and generalization. Indicators such as $TT_{m}$, $DET_{m}$, and $LAM_{m}$ exhibit very low RMSE, NRMSE and MAE values, indicating excellent local fit and minimal prediction error. These models are well-suited for precise estimation within their respective domains. The normalized root mean square error was used to assess model performance independently of the scale of the analyzed variables. It provides a dimensionless measure of error, allowing for direct comparison across different indicators. Lower NRMSE values indicate better model fit, with values below 0.1 typically considered excellent. In this study, NRMSE varied considerably across parameters, reflecting differences in data variability and model sensitivity. This suggests that the best-fitting are $TT_{m}$, $DET_{m}$ and $ENT_{m}$.

In contrast, $L_{mmax}$, $T1_{m}$, and $T2_{m}$ show significantly higher RMSE and MAE values, which can be attributed to the larger scale of the analyzed variables. However, their normalized RMSE values remain within acceptable bounds, suggesting that the absolute error is proportional to the magnitude of the data. The determination coefficients $R^{\prime 2}$ are generally high across most indicators, with $T1_{m}$ and $T2_{m}$ exceeding 0.9, confirming strong explanatory power. Adjusted $R^{2}$ values also support this, although $DET_{m}$ and $TT_{m}$ show relatively low adjusted scores (0.0123 and 0.2256), indicating limited generalization despite low error metrics. Overall, the models provide reliable fits for most indicators, especially those with consistent scale and structure.

The analysis of the average derivative of $RQA_{m}\left(v_{s},v_{f}\right)$, as defined in Equation (4), facilitates the identification of the dominant grinding parameter by evaluating the mean absolute values of the partial derivatives with respect to $v_{s}$ and $v_{f}$ across the examined range:

(5) $$\frac{\partial RQA_{m}\left(v_{s},v_{f}\right)}{\partial v_{s}}=\frac{1}{n}\sum_{i=1}^{n}\left|p_{10}+2p_{20}v_{s}^{\left(i\right)}+p_{11}v_{f}^{\left(i\right)}\right|,$$

(6) $$\frac{\partial RQA_{m}\left(v_{s},v_{f}\right)}{\partial v_{f}}=\frac{1}{n}\sum_{i=1}^{n}\left|p_{01}+2p_{02}v_{f}^{\left(i\right)}+p_{11}v_{s}^{\left(i\right)}\right|,$$

*n* represents the total number of data points considered in the analysis.

As shown in Equation (7), the dominant index (DI) is computed by assessing the difference between the partial derivatives given in Equations (5) and (6), thereby providing a quantitative measure of the relative impact of cutting speed and feed rate.

(7) $$DI\left(RQA_{m}\right)=\frac{\partial RQA_{m}\left(v_{s},v_{f}\right)}{\partial v_{f}}-\frac{\partial RQA_{m}\left(v_{s},v_{f}\right)}{\partial v_{s}}.$$

This indicator facilitates the evaluation of whether cutting speed ($v_{s}$) or feed rate ($v_{f}$) has a more significant impact on the selected recurrence metrics. A positive value of DI indicates that $v_{f}$ is the dominant factor, while a negative value suggests that $v_{s}$ exerts greater influence. Values of DI close to zero imply that both parameters contribute with comparable significance.

**Figure 14:**
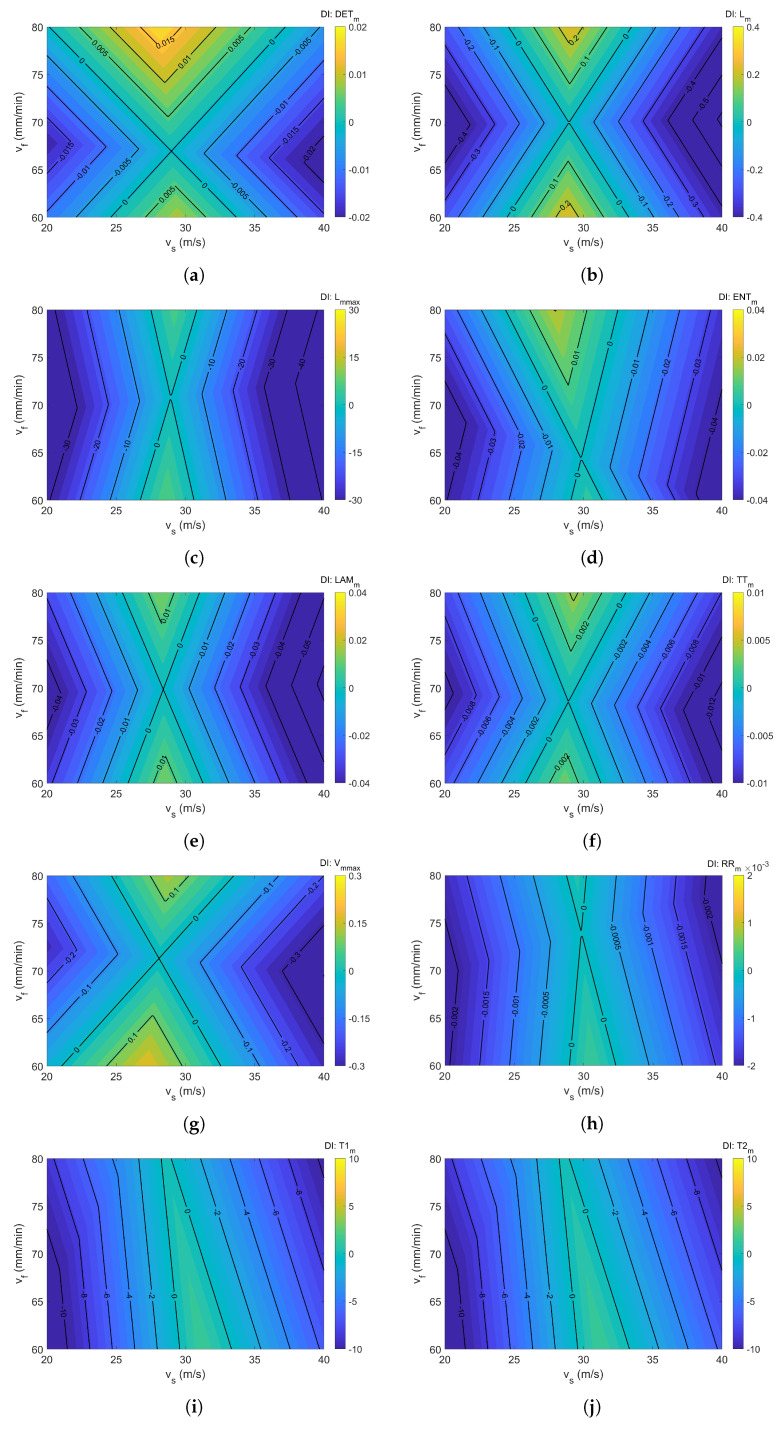
Domination indexes for: (**a**) $DET_{m}$, (**b**) *L*, (**c**) $L_{mmax}$, (**d**) $ENT_{m}$, (**e**) $LAM_{m}$, (**f**) $TT_{m}$, (**g**) $V_{mmax}$, (**h**) $RR_{m}$, (**i**) $T1_{m}$, and (**j**) $T2_{m}$. The colorbar represents the magnitude of the DI.

Figure 14a–j depict the dominant index values associated with individual recurrence indicators. The dimensionless nature of the DI index allows for relative comparison, indicating which parameter exerts a stronger influence on the selected $RQA_{m}$ measure. To enhance the interpretability of the plot, a filled contour representation with DI isolines was used. Blue areas indicate negative DI values, while yellow and green correspond to positive ones. Yellow-highlighted regions mark areas where the feed rate exerts a stronger influence than grinding speed on the $RQA_{m}$ metrics. A comprehensive analysis across all indicators reveals a consistent conclusion: cutting speed has the most pronounced effect on recurrence measures, as indicated by the predominance of blue-shaded regions. A localized dominance of feed rate is observed only around a grinding speed of 30 m/s, aligning with the previously presented RQA surface plots. This outcome is attributable to the presence of stability lobes and chatter phenomena, which define the boundaries between stable and unstable cutting regimes [41].

It is noteworthy that $DET_{m}$ (Figure 14a) demonstrates the greatest sensitivity among all indicators, as evidenced by the broad yellow-highlighted areas. Following closely are $L_{m}$ (Figure 14b) and $V_{mmax}$ (Figure 14g), which also show considerable responsiveness. Interestingly, some indicators exhibit greater sensitivity to the feed rate $v_{f}$ than others. Indicators $T1_{m}$ (Figure 14i), $T2_{m}$ (Figure 14j), and $RR_{m}$ (Figure 14f) show low responsiveness to feed rate but react more strongly to cutting speed. The recurrence time indicators display nearly identical dominance index maps, as seen in the T1 and T2 surfaces (see Figure 13b,c), suggesting a consistent influence pattern. The dominance index DI maps for $L_{m}$ (Figure 14b) and $LAM_{m}$ (Figure 14e) are notably symmetric with respect to feed rate and cutting speed. This symmetry implies that laminar states undergo similar changes with increasing feed rate across the entire range of cutting speeds. A comparable symmetry is also observed for $TT_{m}$ (Figure 14f), $V_{mmax}$ (Figure 14g), and $L_{mmax}$ (Figure 14c). In contrast, the least symmetric DI maps are associated with $DET_{m}$ (Figure 14a), $ENT_{m}$ (Figure 14d), $RR_{m}$ (Figure 14h), and the recurrence time indicators $T1_{m}$ (Figure 14i) and $T2_{m}$ (Figure 14j). This asymmetry suggests that near the critical grinding speed, the dominance of cutting speed ($v_{s}$) may increase significantly.

To summarize, the DI maps reveal that cutting speed tends to exert a greater impact on the RQA measures overall. Nevertheless, for specific parameters, the feed rate may also play a dominant role.

## 6. Conclusions

This study presents a comprehensive analysis of grinding forces dynamics during end mill machining. Due to the inherent complexity of the grinding process, this area remains relatively underexplored in the existing literature. In the experimental phase, grinding tests were conducted under varying cutting speeds and feed rates. Based on the collected data, a mathematical model describing the grinding force was developed. Additionally, recurrence diagrams were generated and analyzed using recurrence quantification analysis.

The results showed that during grinding with a 1A1-type diamond wheel, the single-pass straight flute of the TSF22 ultrafine grained cemented carbide end mill experienced an average cutting force of approximately 200 N, with peak values reaching up to 250 N. These values were observed at cutting speeds ranging from 20 to 40 m/s and feed rates between 60 and 80 mm/min.

A similar conclusion was drawn from the recurrence plots: higher cutting speeds resulted in more periodic system dynamics, characterized by clearer diagonal structures and more dominant frequency components. The influence of feed rate on the RP structure was relatively minor; the main diagonal remained largely unchanged, with only additional recurrence points appearing. Interestingly, at a feed rate of 70 mm/min, the recurrence plots exhibited the most periodic structure. The RQA results confirmed that the analyzed signals generally exhibit predominantly deterministic behavior, as indicated by high values of the DET metric. Most recurrence indicators based on diagonal line structures reached their maximum values at a cutting speed of 30 m/s and a feed rate of 70 mm/min, suggesting that the system demonstrates the most stable and periodic behavior under these conditions (i.e., for the TSF22 tool, 1A1 grinding wheel, and within the analyzed parameter range). These findings support the recommendation of 30 m/s as the optimal cutting speed for end mill grinding.

Notably, the ENT parameter did not fully corroborate this conclusion. Recurrence measures based on vertical line structures, such as LAM and $V_{max}$, also attained their highest values at a cutting speed of 30 m/s. Similar trends were observed for RR and the recurrence time metrics T1 and T2. Among all indicators, $V_{max}$, $L_{max}$, and ENT appeared to be the most sensitive to variations in cutting parameters, as evidenced by their highly complex surface profiles. In contrast, TT exhibited definitely lower sensitivity.

Based on the obtained RQA surface maps, nonlinear mathematical models of recurrence quantification were proposed. This modeling approach enables accurate prediction of system behavior without the need for extensive experimental procedures, which is particularly advantageous given the time and cost constraints associated with grinding process studies.

To evaluate which cutting parameter exerts a greater influence on recurrence measures, a new metric, referred to as the dominant index (DI) was introduced. This index enables a comparative assessment of the relative impact of cutting speed and feed rate on the selected recurrence-based indicators. The DI maps revealed that, overall, cutting speed is the more dominant factor within the analyzed parameter space. However, near a cutting speed of 30 m/s, feed rate emerged as the more sensitive parameter. Symmetry observed in the DI maps for indicators such as LAMm, TTm, $V_{mmax}$, and $L_{mmax}$ suggests that feed rate sensitivity peaks around $v_{f}\approx 70$ mm/min (notably, the RP diagrams for this setting were also more periodic). In contrast, asymmetry in the maps for DETm, ENTm, RRm, T1m, and T2m indicates that this value is slightly shifted.

The results obtained can be used to assess the stability of the chip groove grinding process and to avoid operating within hazardous parameter ranges. Additionally, the proposed mathematical models allow for the estimation of force values and recurrence indices, thereby improving grinding efficiency. These findings may also contribute to the detection of grinding wheel breakage, which remains one of the most critical challenges in this type of machining.

## Figures and Tables

**Table 1:** Mechanical and physical properties of the ground carbide TSF22 [16].

Grade	Classification	Grain Size μm	Co %	Density g/cm^3^	Hardness HV30	Transverse Rupture Strength MPa
TSF22	ultrafine	0.2–0.5	8.2	14.5	1930	4400

**Table 2:** Grinding process parameters and test conditions.

Parameters/Flute Geometry		Grinding Process	
Grinding width ($a_{e}$)	3.00 mm	Coolant	grinding oil
Grinding depth ($a_{p}$)	5.21 mm	Grinding method	single-pass grinding
Cut layer cross-sectional (p)	11.21 mm^2^	Type of machining	climb grinding
Number of flutes (z)	4	Grinding wheel	type 1A1 (diamond wheel)
Helix angle (λ)	0°	Wheel diameter ($D_{s}$)	100 mm
Rake angle (γ)	0°	Wheel width (*U*)	10 mm
Grinding length (*l*)	30 mm	Workpiece	
Pitch	equal	Workpiece diameter ($D_{p}$)	12 mm
Flute	straight	Material	cemented carbide (TSF22)

**Table 3:** Configuration of cutting parameters used in grinding tests.

No.	1	2	3	4	5	6	7	8	9
$v_{f}$ (mm/min)	60	60	60	70	70	70	80	80	80
$v_{s}$ (m/s)	40	30	20	40	30	20	40	30	20

**Table 4:** Characterization and definition of recurrence measures [21,25,30,31,32,33,34]. P(l) and P(v) are the histograms of the diagonal or vertical structures, $N_{l}$ and $N_{s}$ are the total number of diagonal lines.

RQA	Notation	Equation	Description
Measures based on diagonal lines			
Determinism	DET	$\frac{\sum_{l=l_{\min}}^{N}lP\left(l\right)}{\sum_{i,j}^{N}R_{i,j}^{\epsilon }}$	Fraction of recurrence points forming diagonal structures
Average Length	L	$\frac{\sum_{l=l_{\min}}^{N}lP\left(l\right)}{\sum_{l=l_{\min}}^{N}P\left(l\right)}$	Mean length of the diagonal lines in RP
Longest line	L_max_	$\max({\left\{l_{i}\right\}}_{i=1}^{N_{l}})$	Maximal length of the diagonal lines in the RP
Entropy	ENTR	$-\sum_{l=l_{\min}}^{N}p\left(l\right)\ln p\left(l\right)$	Probability distribution of the diagonal line
Measures based on vertical lines			
Laminarity	LAM	$\frac{\sum_{v=v_{\min}}^{N}vP\left(v\right)}{\sum_{v=1}^{N}vP\left(v\right)}$	Fraction of recurrence points forming vertical structures
Trapping Time	TT	$\frac{\sum_{v=v_{\min}}^{N}vP\left(v\right)}{\sum_{v=v_{\min}}^{N}P\left(v\right)}$	Mean length of the vertical lines in RP
Longest line	V_max_	max$\left({\left\{v_{l}\right\}}_{l=1}^{N_{v}}\right)$	Maximal length of the vertical lines in the RP
Measures based on points, distances and times			
Recurrence Rate	RR	$\frac{1}{N^{2}}\sum_{i,j=1}^{N}R_{i,j}^{\epsilon }$	Density of recurrence points
Recurrence Time	T1	$\min\left\{v_{i}\in N|v_{i}\geq v_{\min}\right\}$	Vertical distance in RP
Recurrence Time	T2	$\max\left\{v_{i}\in N|v_{i}\geq v_{\min}\right\}$	Vertical distance in RP

**Table 5:** The statistical parameters for the normal force component (based on data directly exported from the statistical software).

Parameters	St. Dev. SD (N)	Mean y (N)	Variat. co. CV (%)	Deter. co. $R^{\prime 2}$ (−)	Adjus. co. $R^{2}$ (−)	Predic. co. $R_{p}^{2}$ (−)	Sig. No. Rat. SNR (−)
Value	6.09	194.08	3.14	0.972	0.962	0.937	29.98
*p*-values/F-values of the regression coefficients							
Parameters	$v_{f}$		$v_{s}$		$v_{f}v_{s}$		
*p*-values	<0.0001		<0.0001		0.0143		
F-values	64.05		211.39		9.72		

**Table 6:** Parameters of recurrence quantification models.

Recurrence Measures ${\mathrm{RQA}}_{m}\;\left(v_{s},v_{f}\right)$	$\underset{\left(\text{-}\right)}{p_{00}}$	$\underset{\left(s/m\right)}{p_{10}}$	$\underset{\left(\min/\mathrm{mm}\right)}{p_{01}}$	$\underset{\left(s^{2}/m^{2}\right)}{p_{20}}$	$\underset{\left(s\cdot \min/m\cdot \min\right)}{p_{11}}$	$\underset{\left(\min^{2}/{\mathrm{mm}}^{2}\right)}{p_{02}}$
${\mathrm{DET}}_{m}$	−3.37522	0.07140	0.09778	−0.00107	−0.00014	−0.00070
$L_{m}$	−74.01298	1.56947	1.70972	−0.02803	0.00077	−0.01238
$L_{mmax}$	−3273.19444	119.89167	53.25833	−2.23333	0.13250	−0.40333
${\mathrm{ENT}}_{m}$	−4.65385	0.19209	0.10734	−0.00255	−0.00062	−0.00069
${\mathrm{LAM}}_{m}$	1.65662	0.15825	−0.10141	−0.00270	−0.00007	0.00074
${\mathrm{TT}}_{m}$	2.53888	0.03196	−0.02721	−0.00060	0.00004	0.00019
$V_{mmax}$	37.36111	0.75833	−1.25833	−0.01667	0.00250	0.00833
${\mathrm{RR}}_{m}$	−0.03579	0.00742	−0.00156	−0.00011	−0.00001	0.00001
$T1_{m}$	606.54954	−42.61193	1.19686	0.54419	0.14403	−0.03079
$T2_{m}$	645.67593	−39.82207	−0.38070	0.49138	0.13854	−0.01854

**Table 7:** Error metrics for regression models fitted to $RQA_{m}$ indicators.

${\mathrm{RQA}}_{m}\;\left(v_{s},v_{f}\right)$	RMSE (−)	NRMSE (−)	MAE (−)	$R^{\prime 2}$ (−)	$R^{2}$ (−)
${\mathrm{DET}}_{m}$	0.0562	0.0691	0.0494	0.6296	0.0123
$L_{m}$	0.5734	0.0995	0.4730	0.8758	0.6687
$L_{mmax}$	44.9825	0.2681	36.4259	0.8664	0.6437
${\mathrm{ENT}}_{m}$	0.1072	0.0749	0.0900	0.6617	0.0979
${\mathrm{LAM}}_{m}$	0.0609	0.3064	0.0500	0.8579	0.6211
${\mathrm{TT}}_{m}$	0.0284	0.0140	0.0234	0.5404	0.2256
$V_{mmax}$	0.4937	0.1932	0.4197	0.8205	0.5212
${\mathrm{RR}}_{m}$	0.0018	0.1649	0.0015	0.8946	0.7189
$T1_{m}$	9.4482	0.1079	8.1255	0.9050	0.7467
$T2_{m}$	8.8642	0.0903	7.8697	0.9043	0.7449

## Data Availability

The original contributions presented in this study are included in the article. Further inquiries can be directed to the corresponding author.

## Abbreviations

The following abbreviations are used in this manuscript:

PVD	Physical vapour deposition
CVD	Chemical vapour deposition
RP	Recurrence plot
RQA	Recurrence quantification analysis
MI	Mutual information
FNN	False nearest neighbors
RR	Recurrence rate
DET	Determinism
LAM	Laminarity
ENT	Entropy
TT	Trapping time
DI	Dominant index
FNN	False nearest neighbours
FFT	Fast Fourier transformation
